# Supplementary material for: Are carbon emissions trading and green financial instruments synergistic? -Comprehensive quantitative research based on content analysis
Source: PLoS One. 2024 Mar 7;19(3):e0298601. doi: 10.1371/journal.pone.0298601 (PMC10919601; doi:10.1371/journal.pone.0298601)
Supplement: S1 Data — (ZIP) [file pone.0298601.s001.zip › Data/地级市Z_F工作报告.zip!/╔╜╢1⁄2/▒⌡╓▌╩╨/└┤╫╘╣1⁄2╓┌║┼í╛┬φ┐╦╩2╛▌═°í┐_2008.docx]

各位代表:

　　现在,我代表市人民政府,向大会作政府工作报告,请予审议,并请市政协委员和其他列席会议的同志提出意见。

　　一、过去五年工作的回顾

　　市八届人大一次会议以来,在省委、省政府和市委的坚强领导下,在市人大、政协的监督支持下,在历届领导班子打下的良好基础上,市政府团结依靠全市人民,以邓小平理论和“三个代表”重要思想为指导,以科学发展观统领全局,始终坚持从滨州实际出发,做好结合文章,解放思想,与时俱进,抢抓机遇,务实苦干,按照“坚持‘五个统筹’、建设系统‘九州’”的总体部署,认真执行“30”字工作方针,聚精会神搞建设,一心一意谋发展,“十五”计划胜利完成,“十一五”规划进展顺利,城乡面貌发生巨大变化,人民生活水平明显提高,精神风貌昂扬向上,成功实现了飞机上天、轮船下海、汽车上路、铁路过河等重大目标。预计2007年生产总值1000亿元,是2002年的2.89倍,年均增长17.4%;人均生产总值26720元,是2002年的2.81倍,年均增长16.8%;地方财政收入59亿元,是2002年的4.13倍,按可比口径年均增长37.7%;国税收入(不含海关代征)72.3亿元,是2002年的4.78倍,年均增长36.7%;地税收入35.65亿元,是2002年的3.59倍,年均增长29.14%;三次产业比例由2002年的18.5:50.6:30.9调整为10.2:62.8:27,初步实现了由欠发达到基本告别欠发达的历史性转变。

　　——工业兴市实现新突破。大力实施“工业兴市”战略,以结构调整为主线,以园区建设为载体,以自主创新提升企业核心竞争力为重点,以膨胀骨干企业为抓手,着力培育壮大十大产业(链)集群。预计2007年规模以上工业增加值540亿元、利税200亿元、利润130亿元,分别是2002年的3.9倍、6.9倍和7.8倍,年均分别增长31.2%、47.3%和50.7%。规模以上企业达到1253家,比2002年增加869家,其中主营业务收入过100亿元的达到4家。工业增加值占GDP的比重由2002年的44.7%提高到2007年的57.3%。高新技术产业产值达到300亿元,是2002年的21倍。中国名牌产品达到14个、驰名商标4件,山东名牌产品73个、著名商标42件。被认定国家级产品实验中心1个、国家级技术中心3个。节能减排工作取得明显成效,完成了省下达的各项指标任务。

　　——民营经济和服务业开创新局面。放宽市场准入,加大扶持力度,民营经济得到长足发展,预计2007年完成增加值625亿元,是2002年的4.29倍,年均增长24.8%;实缴税金69亿元,是2002年的11.7倍,年均增长61.2%。大力发展现代物流、文化旅游、会展经济等新兴产业,现代服务业快速发展,预计2007年实现增加值270亿元,是2002年的2.53倍,年均增长16%;从业人员达到61.6万人,比2002年增加10.5万人,占全社会从业人员比重达到28.7%;旅游总收入20.17亿元,是2002年的2.96倍,年均增长24.2%。

　　——城乡规划建设展现新面貌。加强城市规划建设管理,滨州新区和经济开发区建设初具规模,高标准完成了“四环五海”工程,初步搭起了生态园林型现代城市框架。“中海”被命名为“国家级水利风景区”。我市被命名为“全国水土保持与生态建设示范市”,被省政府授予“山东省适宜人居环境奖”称号。新立河、秦台河、张肖堂干渠综合治理基本完成。中心城区建成区面积、城市人口分别达到74.3平方公里和49.5万人,城区人均道路、绿地、住房面积大幅增加。县城、中心镇建设稳步推进,一大批小城镇快速发展,全市已拥有国家重点镇3个和省级中心镇9个。

　　——基础设施建设进入新阶段。五年间完成投资2100亿元,是改革开放以来到2002年24年投资总和的3.65倍。我市被确定为国家级交通运输主枢纽。滨博高速、滨大高速及荣乌、津汕高速公路滨州段竣工通车,滨博高速公路黄河大桥、惠青黄河大桥、滨州公铁黄河大桥建成通车。套尔河大桥开工建设。滨港铁路一期工程实现试运行。黄河二期标准化堤防建设进展顺利。滨州万吨级大港导堤工程稳步推进。大高航空城、滨州西客站建设进展顺利。城市主干道、出入口景观焕然一新,一大批基础设施工程建成使用,成为滨州新的风景线。

　　——新农村建设跃上新水平。全面落实支农惠农政策,严格依法保护耕地,新农村建设全面展开。全市财政对“三农”累计投入47.07亿元,年均增长25.6%。建成市级以上龙头企业175家,标准化生产基地150万亩。预计2007年农业总产值200亿元,比2002年增长44.2%,年均增长7.6%。森林覆盖率达到25.4%,比2002年提高8.3个百分点。总蓄水能力达到7.5亿立方米,比2002年增加4.1亿立方米;农村自来水普及率由30%提高到95.6%;基本实现了村村通柏油路,100%的乡镇和95%的行政村实现了通客车;村村通有线电视率达到100%;新农合参合率达到88.64%。——各项改革迈出新步伐。农村综合改革取得明显成果,农业税、农业特产税及涉农收费全部减免,各项补贴得到落实。国有企业和国有资产监管体制改革取得重要进展。粮食、棉花流通体制改革顺利完成。农村义务教育“以县为主”管理体制改革、经费保障机制改革等取得显著成效。机关事业单位人事、收入分配制度改革取得新突破。投资、财税、住房、医疗卫生、土地等改革稳步推进。行政审批制度改革不断深化,服务水平明显改善。

　　——对外开放再创新业绩。坚持对外开放与对内开放相结合,大力发展开放型经济。2007年全市进出口预计完成33.5亿美元,是2002年的4.35倍。五年累计引进市外资金1792.22亿元,其中境外资金16.2亿美元,年均增长56%。新增境内外上市公司5家,全市已达到7家,累计融资87亿元,境外上市工作走在了全省前列。新增商业银行1家。连续举办了六届银企合作推进会,签约资金达2920.6亿元,前五届资金到位率达到95.6%,2007年合同资金709.6亿元,协议资金425.4亿元。滨州经济开发区从小到大、由弱变强,各项事业迅速发展,基础设施进一步完善,大高通用航空高新技术产业开发区已经省政府批准,全市省级经济开发区达到9家,为加快对外开放提供了坚实载体。

　　——县域经济发展呈现新格局。认真落实县域经济发展和“对口帮扶”政策,大力实施“渡河战役”,促强、扶弱、带中间,邹平成为全国百强县,无棣在全省30个弱县中经济增长名列前茅,阳信、惠民经济增长速度明显加快,博兴、滨城、沾化综合经济实力进一步增强,南北县区经济协调发展。高起点谋划和实施“北带”开发,建成防潮大堤210公里,新增土地63万亩,沿海生态科技产业快速崛起。“北海新区”规划建设开始启动。

　　——和谐社会建设取得新成就。深入实施“科教兴滨”战略,2007年全市应用技术研究与开发资金达到7270万元,是2002年的3.7倍,五年累计申请专利3132件;40万名城乡义务教育阶段学生告别交杂费历史,初步建立起家庭贫困学生资助体系;五年间共向高校输送本科学生3.5万名;新建本科院校1所,高等职业院校2所;人才队伍建设成效明显,人才结构进一步优化;文化事业蓬勃发展,竞技体育实现历史性跨越;抗击“非典”取得重大胜利;疾病预防控制和医疗救治体系建设任务顺利完成;人口计生工作新机制建设深入推进,人口责任目标圆满实现;顺利通过了国家卫生城市复核验收;成功创建了国家级双拥模范城。扎实开展省级文明城市及群众性文明创建工作,市民的文明素质不断提高。社会保障体系建设进一步加强,出台了城乡低保、五保供养、灾民救助、住房、医疗等十项救助制度,全市城乡10.7万名低保人群实现了应保尽保;城镇新增就业再就业21.8万人,城镇“零就业”家庭和90%的农村“零就业”家庭实现了每户至少一人就业;经济适用房建成面积180万平方米,廉租住房制度顺利实施。深入开展“平安滨州”建设,狠抓社会治安综合治理,高度重视信访工作,全面落实安全生产责任制,大力实施食品药品放心工程,防灾减灾工作卓有成效,社会秩序和谐稳定。民族宗教、妇女儿童、新闻出版、外事侨务、对台、油区管理、物价、残疾人事业、档案、史志、老龄、地名、地震、海事、人防、气象等工作都有了新的发展。

　　——人民生活水平迈上新台阶。城乡居民收入增加较快,衣食住行用水平不断提高。预计2007年城镇居民人均可支配收入达到13800元、农民人均纯收入4900元,分别比2002年增长87.2%和83.5%,年均分别增长13.4%和12.9%。预计2007年城乡居民人均储蓄达到7811元,比2002年增长70.3%,年均增长11.2%。

　　——政府自身建设得到新提升。坚持依法行政,制定完善了一系列财政、人事制度,坚持用制度管人、用制度管事、用制度管钱,坚持公开透明、阳光操作,政府决策的民主化、科学化水平进一步提高。全面贯彻实施《行政许可法》和《全面推进依法行政实施纲要》,大力推行政务公开、厂务公开、村务公开。认真办理市人大常委会审议及视察意见、人大代表建议和政协委员提案。坚持不懈地开展廉政建设和反腐败斗争,加强领导干部经济责任和政府投资审计,严肃查处违法违纪案件。市公共行政审批服务中心、市长公开电话受理中心、市级政府采购中心工作进一步完善。政府应急管理体制启动运行。投资建设了市级电子政务平台,开通了政府门户网站,免费发行了《滨州市人民政府公报》,促进了阳光政府建设。

　　2007年,市政府和全市人民一道,深入贯彻落实党的十七大和省、市党代会精神,加快推动工作指导转变,积极应对国家宏观调控政策,深入贯彻落实科学发展观,按照“五个加快推进”和“五个更加注重”的要求,紧紧抓住发展第一要务,全力推动产业链延伸、科技进步、港口建设、铁路运营、节能环保绿化、城乡平安和谐稳定、解决城乡家庭零就业等重点工作,全面落实中央、省、市出台的一系列改善民生的政策措施,“奋力拼搏2007,科学发展再创佳绩”,完成了市八届人大五次会议确定的各项任务。

　　各位代表!过去的五年,是滨州历史上经济发展最快、面貌变化最大的五年,是综合实力大幅增强、人民生活水平大幅提高的五年。这是省委、省政府和市委正确领导的结果,是人大、政协和社会各界监督、关心、支持的结果,是全市人民不懈奋斗的结果!在此,我代表市政府,向辛勤工作在各条战线的全市人民群众,向各位人大代表、政协委员,向各民主党派、工商联、各人民团体和各界人士,向中央和省驻滨单位,向驻滨人民解放军、武警官兵和公安干警,向来滨的所有投资者和外来劳动者,向所有关心支持滨州发展的海内外各界朋友,表示衷心的感谢!

　　同时,我们也清醒地看到,全市经济总量不大、结构不够合理的局面还没有根本转变;经济增长方式粗放、创新能力不强、南北发展不平衡的矛盾仍然比较突出;重大基础设施建设、现代服务业的发展仍是薄弱环节;社会保障体系不够健全,社会保障能力不强,人民总体生活水平不够高,还存在一些不稳定、不和谐的因素;环境保护、节能降耗、新农村建设的任务还十分艰巨。政府公共服务水平不够高,发展环境不够宽松;观念不新、效率不高、作风不实、推诿扯皮,甚至以权谋私、贪污腐败等现象还依然存在。对以上问题,我们必须高度重视,采取得力措施认真加以解决。

　　二、今后五年目标任务和总体要求

　　今后五年,是滨州全面建设小康社会的关键时期。随着省委、省政府“一体两翼”区域经济发展战略的全面实施,滨州作为“北翼”开发的主战场,面临的机遇前所未有,面临的挑战也前所未有。全面推进重大基础设施建设,进一步关注民生、改善人民生活仍然是我们的重大任务。按照市七次党代会提出的目标任务,继续推动滨州改革开放和现代化建设进程,是各级政府义不容辞的历史使命。今后五年政府工作的总体要求是:高举中国特色社会主义伟大旗帜,以党的十七大精神为指导,深入贯彻落实科学发展观,继续执行“30字”工作方针,坚持好字优先,稳中求快,坚持解放思想,改革开放,夯实基础,埋头苦干,调整提升,巩固完善,围绕建设民富市强、文明和谐的现代滨州,全面实施工业兴市、科技强市、三产活市、生态立市、统筹富市“五大战略”,着力推进全市经济社会又好又快发展,在新起点上实现新跨越。

　　主要发展目标是:人均生产总值年均增长15%,到2012年比2007年实现翻番,到2020年力争实现再翻番;地方财政收入年均增长20%;城镇居民人均可支配收入、农民人均纯收入年均增长10%以上;单位生产总值能耗和主要污染物排放总量完成省下达任务指标;人口自然增长率控制在6.5‰以内。经过五年的努力,全市综合经济实力进一步增强,地方财力进一步壮大,城乡面貌明显变化,生态环境明显改善,人民生活水平明显提高,文化更加繁荣,社会更加和谐,力争提前全面建成小康社会,让现代滨州焕发出更加蓬勃的生机和活力!

　　(一)坚定不移地推进经济发展方式转变。积极促进经济增长由主要依靠投资、出口拉动向依靠消费、投资、出口协调拉动转变,由主要依靠第二产业带动向依靠第一、第二、第三产业协同带动转变,由主要依靠增加物质资源消耗向主要依靠科技进步、劳动者素质提高、管理创新转变。继续加大投入,进一步优化投资方向、突出投入重点,紧紧围绕先进装备制造业、现代服务业、高新技术产业、基础设施、新农村建设等,建成一批重点项目和重大工程,夯实发展基础,增强发展后劲。实施“工业兴市”战略不动摇,大力延伸十大产业链,加大研发和技改力度,积极培育大型企业集团和名牌产品,提高企业核心竞争力,尽快形成产业集群优势。加快发展现代服务业,进一步激活创业主体,创新体制机制,大力发展现代物流、商贸流通、会展、旅游、中介、房地产、金融担保保险、文化体育、餐饮娱乐等新兴产业。加快“北海新区”规划建设,以万吨大港建设为龙头,大力发展临港产业和海洋经济,培育沿海特色产业集群,着力打造高效生态现代工业园区,积极推进与天津滨海新区、省会城市群经济圈、半岛城市群产业对接,促进滨州科学发展、超越发展。

　　(二)坚定不移地推进可持续发展。全面提高城乡规划建设管理水平,科学完善中心城市规划,优化城乡规划布局,统筹新老城区建设,积极推进县城和中心镇建设,加快市政公用事业产业化步伐,完善城市功能,提升城市品位,提高城市管理执法和服务水平,增强辐射带动能力。突出加快交通基础设施建设,全力推进高速公路、港口、铁路、航空城等重大工程建设步伐,建设公路、铁路、海上运输、城市快速干道相配套的现代交通体系。积极推进社会主义新农村建设,坚持以工促农、以城带乡、典型示范,大力发展现代农业,引导农民发展二三产业,千方百计增加农民收入。积极推进“生态文明”建设,在全社会牢固树立生态文明观念,大力发展循环经济,以节能、节地、节水、节材和资源综合利用为重点,抓好节能降耗工作;合理开发、集约利用土地资源;深入实施“蓝天碧水工程”,加大治污减排力度,积极创建国家环保模范城市;全面推进生态市建设,切实改善城乡生态环境。

　　(三)坚定不移地推进以改善民生为重点的社会建设。全面繁荣社会各项事业,优先发展教育事业,深入实施素质教育,促进义务教育均衡发展,加快普及高中阶段教育,大力发展职业教育,提高高等教育质量;加快建设覆盖城乡的公共卫生服务体系,扎实推进新型农村合作医疗制度,积极推进城市社区卫生服务;开展全民健身运动,发展体育事业,办好第11届全运会滨州分赛场比赛;稳定低生育水平,努力优化人口结构,统筹解决人口问题。全面提高人民群众生活水平,健全完善社会保险、社会救助、社会福利等保障体系,加强城乡低保、农村五保和困难群众救助,完善企业职工和农民工工资保障长效机制;进一步建立健全廉租房制度,改进规范经济适用房制度,切实解决城市低收入家庭住房困难;更加重视就业再就业工作,建立健全市县乡三级公共就业管理服务体系。全面提升“平安滨州”建设水平,强化矛盾纠纷排查调处;发展和规范法律服务,高度重视法律援助工作,促进社会公平正义;大力整顿和规范食品药品市场秩序,确保人民饮食用药安全;严格安全生产管理和监督,有效遏制各类安全事故发生,全力维护社会稳定。全面提高依法治市水平,进一步推行依法行政;加强基层政权建设,健全充满活力的基层群众自治机制;搞好社区建设和管理,打造管理有序、服务完善、文明和谐的新型社区。

　　(四)坚定不移地推进社会主义文化建设。坚持社会主义先进文化前进方向,掀起社会主义文化建设新高潮,切实增强文化软实力。积极建设社会主义核心价值体系,大力弘扬爱国主义、集体主义、社会主义思想,加强社会公德、职业道德、家庭美德、个人品德建设,用社会主义荣辱观引领社会风尚。加大文化体育等基础设施投入力度,启动市民文化中心、奥林匹克公园建设。深化文化体制改革,大力发展文化事业和文化产业,积极培育壮大文化骨干企业。积极开展群众性文化活动,培育和发展社区文化、乡村文化、企业文化。深入挖掘整合滨州丰富的文化资源,做大做强黄河文化、孙子军事文化、董永孝文化、范仲淹忧乐观文化、“老渤海”革命文化等文化品牌,振兴吕剧等传统戏剧,建设滨州特色文化体系。全面完成各县区第二轮修志。着力抓好广播电视“村村通”、文化信息资源共享、社区和乡镇综合文化站及基层文化阵地建设、农村电影放映、农家书屋建设“五大工程”,努力建设文化强市。

　　三、今年政府工作的重点

　　2008年是全面贯彻落实党的十七大战略部署的第一年,是实施“十一五”规划承上启下的一年。初步确定2008年主要预期目标是:地区生产总值增长15%;地方财政收入增长20%;全社会固定资产投资增长20%;城镇居民人均可支配收入和农民人均纯收入增长10%;城镇居民登记失业率控制在3.8%以内。主要约束性指标是,万元地区生产总值能耗下降4.5%;二氧化硫排放量减少8.4%;化学需氧量排放量降低3.6%;人口自然增长率控制在6.5‰以内。切实抓好十大重点工作。

　　(一)继续抓好“工业兴市”。把加快十大产业链延伸作为全市经济工作的中心,进一步做大产业、做强企业、做优品牌。加大骨干企业培育力度,抓好20个重点工业园区、305家投资过千万元的重点工业项目、100户重点工业企业发展,促进140家成长型中小企业做大做强,力争规模以上工业增加值增长20%。加快发展高新技术产业,用高新技术改造提升传统优势产业,大力发展电子信息、生物制药、先进装备制造业等新兴产业,建立完善企业自主创新激励机制,不断增强企业核心竞争力。年内认定省级高新技术企业10家以上,认定省级工程技术中心2家以上,争创省级重点实验室1家,高新技术产业产值力争达到400亿元以上,占规模以上工业总产值的比重提高2个百分点。年内启动国家生态纺织品暨棉花质量监督检验中心建设。争创中国驰名商标1件,中国名牌产品2个,国家免检产品3个。

　　(二)突出发展现代服务业。把加快服务业发展放到更加重要的位置,积极推进重点城区、重点园区和重点企业“三大载体”建设。抓好市区、各县城、鲁北大街、滨博大道等10个重点城区建设;抓好滨州现代物流中心园区、惠民孙子文化旅游园区、滨州中海景区综合区等20个重点服务业园区建设;抓好滨州客运总站、滨州国际纺织商贸城、奥林匹克公园、滨州火车站、京博物流中心等30个重点服务业项目建设。加快发展旅游业,争创中国优秀旅游城市。加快健全农村现代流通服务“一个网络”,强化农村社区服务中心、农村合作经济组织联合会“两个平台”,全面实施“万村千乡”市场工程建设。大力发展第三方和第四方物流。突出发展生产性服务业,继续抓好大中型企业二三产业分离工作,进一步壮大地方财力。力争服务业增加值实现300亿元以上,比上年增长20%。

　　(三)切实保障和改善民生。进一步加强城乡家庭零就业动态管理,建立完善促进就业的长效机制;实现城镇新增就业3万人,城镇下岗失业人员再就业1.1万人,新增农村劳动力转移5万人。争取列入城镇居民医疗保险试点市。完善劳动保障监察和劳动争议调处仲裁机制。强化社会保障基金、住房公积金等社会公共基金的监督管理。投资4600万元资助普通高中、中等职业学校和普通高校家庭经济困难学生完成学业。投资1.2亿元,在全部免除学生杂费的基础上,提高预算内生均公用经费标准,提高免费教科书和寄宿生生活费补助比例。改造农村中小学危房2.4万平方米。年内在市区新建2所义务教育学校。积极推进城镇水、电、暖、气等基础设施配套建设,着力提高公共服务水平和居民生活质量。提高城市低保补助水平,全市人均补差不低于全省平均水平。加快廉租房和经济适用房建设。规范完善农村低保制度,实现动态管理下的应保尽保。落实五保财政供养政策,符合条件的农村五保对象全部纳入保障范围。加强敬老院建设管理,集中供养率巩固稳定在70%以上,对90岁以上无固定收入老人实施补贴。

　　(四)扎实推进社会主义新农村建设。切实保障公共财政向“三农”倾斜,市级财政安排新农村建设资金1.3亿元,增长45%。大力推进城乡统筹,继续搞好农村“水电路医学”基础设施建设,抓好320个新农村示范村建设。加快培育壮大富民主导产业,带动龙头企业发展、农民增收、劳动力转移和农村产业结构调整,年内规模以上龙头企业达到470家,重点专业合作社发展到30家。加快农机化步伐,重点推广玉米机收和保护性耕作技术。大力推进农业标准化生产。培训农村劳动力10万人以上。加快畜禽规模饲养生产基地建设。抓好重大动植物疫病防控,完善农产品质量检测体系。加大近海资源养护力度,增殖放流渤海湾优势水产品种。大力推进农业综合开发,切实打好农田水利基本建设和植树造林战役,投资5.6亿元,开工和续建各类水利工程2097项,新增蓄水能力4000万方,村村通自来水率达到98%,新建方田30万亩,治理水土流失面积76.7平方公里;投资2.5亿元,加快推进黄河二期标准化堤防工程建设;抓好绿色通道、高酸苹果基地、农田林网、绿化示范村建设,完成造林面积16.5万亩,森林覆盖率增长1.5个百分点。

　　(五)全力抓好基础设施建设。打好交通基础设施建设战役,年内开工建设滨德高速、孙武黄河大桥、疏港路;套尔河大桥完成主桥工程;永莘路滨州段、永馆路沾化至205国道段年底建成通车;完成205线黄河大桥至柳桥段改造;投资7.6亿元改造农村公路1600公里;西客站完成投资2亿元,年内实现运营;加快推进滨州港15公里引堤主体建设,实施防波堤前期工程,加快航道疏浚和内河码头泊位建设;加快铁路一期工程运营步伐;做好滨港铁路二期、黄大、德龙烟铁路前期工作。加强城市规划建设管理执法工作,提高城市经营水平;加大城市建设投入力度,初步安排投资3.58亿元,实施新区建设及老城改造项目53个;加快城中村改造步伐,以点带面,全面推进。加快滨州经济开发区基础设施建设,进一步提高项目承载能力。“北海新区”完成产业规划和城市规划,正式挂牌成立,吸引大项目尽快落地。打好路域综合治理战役,对高速公路、干线公路、县乡公路两侧路网、水网、林网、线网进行“四位一体”综合治理,切实提高经济效益、社会效益和生态效益。

　　(六)全面深化改革开放。坚持改革的正确方向,大力推进改革创新。深化国有企业改革,完善国有资产管理体制,建立产权明晰、权责明确、充满活力的企业新机制。探索建立行政事业国有资产管理新机制,提高政府资源使用效率。积极推进财政预算制度改革,努力构建公共财政体系。建立规范完善的政府投资评审制度,充分发挥政府投资使用效益。积极推进城市公用事业市场化改革,加快投资主体多元化进程。深化农村产权制度改革、金融改革等各项综合改革,激发农村经济发展活力。深化人事制度改革,继续推进事业单位改革,全面推行法人负责制和人员聘用制。进一步扩大开放,提高开放型经济水平。调整优化进出口产品结构,加快机电产品、高新技术产品和终端产品出口。继续加大招商引资力度,围绕打造十大产业(链)集群,引进一批产业关联度大、技术含量高、辐射带动能力强的重大项目,提升产业发展水平和竞争力。继续抓好银企合作工作,办好第七届银企洽谈会。积极推进金融一条街建设。争取商业银行入驻滨州,申办市级农村合作银行。强力推进企业上市工作,力争新增上市企业3家以上。进一步优化发展环境,支持经济开发区加快发展、率先发展。认真落实扶持县域经济发展的各项政策,加大帮扶阳信、惠民力度,促进县域经济协调发展。

　　(七)下大气力推进节能减排。认真落实《国家环境保护“十一五”规划》,进一步建立和完善节能减排目标责任制,确保完成今年的节能减排任务。扎实开展“节能减排全民行动”,加强能源统计监测,突出抓好工业、交通、建筑等重点行业和56户重点用能企业的节能工作,强化考核监督,狠抓节能执法监察,抓好循环经济和清洁生产,千方百计把重点用能企业的能耗水平降下来。运用新技术、新工艺和新设备改造提升传统产业,加快淘汰落后产能。全面推进建筑墙改与节能,新型墙材应用比例达到100%;节能建筑竣工建筑面积比例达到90%以上;新型墙材生产比例达到80%。进一步加强土地管理和耕地保护,提高土地节约集约利用水平。切实改善城乡人居环境,加强水、大气和土壤污染防治。严格执行国家产业政策,严把新上项目技术、耗能、环境“三关”,严格环评和“三同时”制度。继续开展环保专项行动,深入实施“四环”内环保净化工程,加快兴福、韩店、魏桥等10个区域性污水处理厂建设。搞好滨海滩涂湿地等自然生态系统的管理保护。

　　(八)统筹发展各项社会事业。加大投入,深入实施科技示范工程,组织实施科技成果引进转化工程项目300项以上。加快省级高新区建设步伐。全面落实“以县为主”农村义务教育管理体制,提高义务教育经费保障水平,实现县域内城乡教师同工同酬。提高高等教育、职业教育教学质量,培养更多优秀人才。进一步完善县乡村三级管理网络,切实提高新型农村合作医疗制度试点工作水平,加快推进城市社区卫生服务工作。以社区和乡镇基层文化设施为基础,健全完善公共文化服务体系,推进文化创新,繁荣发展社会科学,创作更多的优秀文学艺术作品,大力开展群众性文化活动。做好文物、非物质文化遗产保护和全国第三次文物普查工作。全面落实人口和计划生育目标管理责任制,圆满完成省下达任务指标。扎实做好全国第11届运动会筹备工作,确保完成比赛场馆建设任务。高度重视油区工作,努力做好民族宗教、新闻出版、对台、侨务、档案、史志、老龄、妇女儿童、残疾人、慈善、人防、气象、防震减灾、红十字会等工作。

　　(九)着力促进平安和谐稳定。自觉接受人民代表大会及其常委会的法律监督和人民政协的民主监督,认真听取民主党派、工商联、无党派人士和各人民团体的意见。深入开展“民主议政日”、“联合接访日”、“社情民意日”等活动,着力解决好征收征用土地、城镇房屋拆迁、企业违法排污以及拖欠农民工工资等突出问题。全面实施“五五”普法规划和“三五”依法治市规划,深入推行行政执法责任制,把执法责任落到实处。广泛开展群众性精神文明创建活动,积极争创省级文明城市。加强军民团结,巩固国家级双拥模范城市。完善应急管理机制建设,加强社会治安综合治理,强力推进防控体系建设,深入开展“严打”整治斗争。严格落实安全生产责任制,强化食品、药品、餐饮卫生监管,保障人民群众生命安全。

　　(十)努力建设服务型政府。更加突出优化服务环境,深化行政管理体制改革,加快推进政企分开、政资分开、政事分开,规范行政行为;加快政府职能转变,强化市场监管、社会管理和公共服务,努力提高行政审批中心、市长公开电话受理中心、政府招标采购中心服务水平。更加突出改进作风,坚持求真务实,苦干实干,不尚空谈,不作表面文章,言必信,行必果,严格目标管理责任制,建立重点工作推进机制,完善重大决策和事项督办制度;加强电子政务和政府门户网站建设,全面推进政府信息公开和公共企事业单位办事公开,逐步实现规范化、制度化。更加突出廉政建设,全面落实党风廉政建设责任制,强化审计监督和行政监察;牢固树立艰苦奋斗、勤俭创业的思想,坚决反对挥霍奢侈、铺张浪费;严格行政责任追究,严厉查处各种名目损害群众利益的行为,对违法违纪案件和腐败分子一查到底,依法严惩。

　　各位代表,党的十七大为我们夺取全面建设小康社会新胜利指明了前进的方向。滨州的未来充满希望,滨州的明天更加灿烂。让我们紧密地团结在以胡锦涛同志为总书记的党中央周围,坚持以邓小平理论和“三个代表”重要思想为指导,深入贯彻落实科学发展观,在中共滨州市委的坚强领导下,坚定信心,振奋精神,众志成城,开拓奋进,为创造滨州人民美好的新生活而努力奋斗!
